# Supplementation of rice husk activated charcoal in feed and its effects on growth and histology of the stomach and intestines from giant trevally, *Caranx ignobilis*

**DOI:** 10.12688/f1000research.27036.2

**Published:** 2021-02-15

**Authors:** Firdus Firdus, Samadi Samadi, Abdullah A. Muhammadar, Muhammad A. Sarong, Zainal A. Muchlisin, Widya Sari, Siska Mellisa, Satria Satria, Boihaqi Boihaqi, Agung Setia Batubara

**Affiliations:** 1 Department of Biology, Universitas Syiah Kuala, Banda Aceh, Aceh Province, 23111, Indonesia; 2 Graduate School of Mathematics and Applied Science, Universitas Syiah Kuala, Banda Aceh, Aceh Province, 23111, Indonesia; 3 Animal Husbandry, Universitas Syiah Kuala, Banda Aceh, Aceh Province, 23111, Indonesia; 4 Department of Aquaculture, Faculty of Marine and Fishery, Universitas Syiah Kuala, Banda Aceh, Aceh Province, 23111, Indonesia; 5 Department of Biology Education, Faculty of Teacher Training and Education, Universitas Syiah Kuala, Banda Aceh, Aceh Province, 23111, Indonesia; 6 Ujung Batee, Center Brackiswater Aquaculture Development, Ujung Batee, Aceh Besar, Aceh Province, 23361, Indonesia

**Keywords:** foveola gastrica, villi, ratio, biometrics

## Abstract

**Background: **Research on supplementing feed with rice husk activated charcoal was carried out to determine the effect of variations in the concentration of rice husk activated charcoal on the growth and histological features of the giant trevally *Caranx ignobilis* intestine.

**Methods:** This study used an experimental method with a completely randomized design consisting of six treatments and four replications, including adding activated charcoal to feed at concentrations of 0%, 1%, 1.5%, 2%, 2.5%, and 3% for 42 days. The measured parameters included daily growth rate (DGR), specific growth rate (SGR), absolute growth rate (AGR), feed conversion ratio (FCR), feed efficiency (FE), survival rate (SR), length of foveola gastrica, width of foveola gastrica, length of intestinal villi, and width of intestinal villi. Data were analyzed statistically using one-way analysis of variance and Duncan’s test.

**Results:** The results showed that supplementing fish feed with rice husk activated charcoal at different concentrations significantly affected the values of DGR, AGR, FCR, FE, SR, length of the foveola gastrica, length of the villous intestine, and width of the villous intestine, but did not significantly affect SGR or foveola gastrica width.

**Conclusion:** The 2% rice husk activated charcoal treatment showed the best results for all parameters.

## Introduction

Giant trevally ( *Caranx ignobilis*) is a commercially valuable fish species widely distributed in Indonesian waters ^1– 3^. This fish is a top predator in coral reef ecosystems with a relatively long predicted age of 25 years and can grow to a size of 165 cm and 87 kg, making giant trevally ( *C. ignobilis*) the largest species in the Carangidae family ^4^. However, fishing continues to increase without conservation efforts, causing the fish population to decline in the last few decades ^5– 7^. Therefore, efforts towards fish domestication are needed to reduce wild fishing. However, several obstacles have been encountered during domestication, such as slow growth of fish due to underdeveloped feed technology in this species.

The development of giant trevally ( *C. ignobilis*) culture is dependent on trash fish (fish considered to have little value and therefore typically discarded whenever caught) because the fish grow faster than when using commercial feed. However, the costs incurred to provide trash fish are relatively expensive and not balanced with the selling price of fish at harvest. In addition, trash fish are not always available and can carry diseases. Therefore, giant trevally ( *C. ignobilis*) feed technology needs to be assessed immediately, through the compilation of appropriate feed ingredients so that it can support the nutritional needs of the fish. One of the technologies that has the potential to be applied is supplementing the feed with activated charcoal.

Activated charcoal is a non-nutritional ingredient that binds toxic substances during digestion to increase intestinal absorption of food that enters the digestive tract ^8^. Activated charcoal can be produced with a variety of ingredients, such as coconut shells ^9, 10^, pine wood powder ^11^, banana peels ^12^, corn stalks ^13, 14^, peanut shells ^14^, rice straw and rice husk ^14, 15^, oil palm stems and shells ^16, 17^, wine stalks ^18^, bamboo ^19^, almond stems and bark ^20^, and durian peel ^21^. Therefore, activated charcoal has good application potential because the resource is easily found and the feed is supplemented with a particular concentration according to the needs of the cultured fish.

High-carbon activated charcoal has the best function, which includes rice husk. Jasman ^22^ reported that rice husk activated charcoal contains 85–95% carbon. In addition, rice husk activated charcoal has an Iod value of 527.8 mg/g, indicating a good quality of absorbent activated charcoal ^23^. Furthermore, rice husks contain 13%–39% ash, 34%–44% cellulose, and 23%–30% lignin ^24^, where the higher the content of hemicellulose, cellulose, and lignin, the higher the amount of activated carbon and the better the quality of the charcoal ^25^.

Several studies have reported applying activated charcoal in fish, such as supplementing activated charcoal in feed on the growth of fugu rubripes ( *Takifugu rubripes*) ^26^, olive flounder ( *Paralichthys olivaceus*) ^27^, Striped catfish ( *Pangasiaodon* sp.) ^28^, nile tilapia ( *Oreochormis niloticus*) ^8^, african catfish ( *Clarias gariepinus*) ^29^, gilthead seabream ( *Sparus aurata*) ^30^, and beluga sturgeon ( *Huso huso*) ^31^. A study on supplementing coconut shell activated charcoal, mangrove wood, rice husk, and oil palm shell in feed on intestinal growth of giant trevally ( *C. ignobilis*) indicated that 2% activated rice husk charcoal had an optimal effect on growth of intestinal tissues ^32^. However, the appropriate concentration to supplement rice husk activated charcoal in feed for giant trevally ( *C. ignobilis*) has not been reported.

## Methods

### Time and site

This study was conducted from April to September 2019 at the Ujung Batee Brackish Aquaculture Fisheries Center, Ministry of Maritime Affairs and Fisheries, Aceh Besar. The activated charcoal was characterized at the Laboratory and Integrated Testing of Gadjah Mada University, Yogyakarta. The gastric and intestinal histological samples were evaluated at the histology laboratory of the Faculty of Mathematics and Natural Sciences, Syiah Kuala University, Banda Aceh Indonesia.

### Charcoal preparation and activation

Rice husk was ground into flour until smooth, and about 500 g was placed in an iron container that was coated with aluminum foil, and burned in a furnace at 400°C for 1 hour. Nitrogen gas was flowed into the furnace to remove oxygen. The temperature was gradually reduced to 30°C for 1 hour. The charcoal was removed from the furnace and filtered through a 40-mesh sieve, and stored in a bottle before activating. A total of 100 g of charcoal was mixed with 400 ml of 0.2 M citric acid. The solution was stirred for 24 hours and filtered through filter paper. The filtered charcoal was washed with distilled water and dried in an oven at 110°C for 24 hours. The activated charcoal was stored in a desiccator before use.

### Feed preparation

The treated feed was prepared from fish meal, rebon shrimp meal, tapioca flour, coconut oil, CaCO _3_, isoleucine, L-tryptophan, and DL-methionine, and premixed with 50% protein feed content. All ingredients were mixed and analyzed for protein content. Subsequently the feed was added to the rice husk activated charcoal at 1%, 1.5%, 2%, 2.5%, and 3% (see Table 1 for feed makeup).

**Table 1.** Feed formulations (g kg ^−1^) with 50% protein content used in the research.

Feed Ingredients (g kg ^-1^)	Feed Formulation (g)					
	Treatment					
	A	B	C	D	E	F
Rebon shrimp meal	430	430	430	430	430	430
Fish meal	350	350	350	350	350	350
Tapioca flour	160	160	160	160	160	160
Coconut oil	5	5	5	5	5	5
CaCo3	5	5	5	5	5	5
Isoleucine	10	10	10	10	10	10
L-Tryptophan	17.5	17.5	17.5	17.5	17.5	17.5
DL-Methionine	17.5	17.5	17.5	17.5	17.5	17.5
Premix	5	5	5	5	5	5
Total (g)	1000	1000	1000	1000	1000	1000
Rice husk activated charcoal (%)	0	1	1.5	2	2.5	3

### Feeding trial

This study used a completely randomized design method with six treatments and four replications. The fish were fed experimental food containing 50% protein twice a day, at 7 am and 5 pm, at 3% of body weight.

A = Treated feed without activated charcoal (control/0%)
B = Feed treated to contain 1% rice husk activated charcoal
C = Feed treated to contain 1.5% rice husk activated charcoal
D = Feed treated to contain 2% rice husk activated charcoal
E = Feed treated to contain 2.5% rice husk activated charcoal
F = Feed treated to contain 3% rice husk activated charcoal

A total of 360 giant trevally ( *C. ignobilis*) juveniles (average weight, 16.47 ± 4.69 g; average length, 9.61 ± 0.71 cm) were collected from local fishermen in Ulee Lheue Village, Banda Aceh City, Indonesia. The fish was acclimatized in the maintenance pond at Ujung Batee Brackish Aquaculture Fisheries Center for 2 weeks.

Juvenile giant trevally ( *C. ignobilis*) were chosen randomly, and the total weight and length of the fish were measured. The fish were distributed into 24 plastic containers (48 × 43 × 70 cm) with a water volume of 75 liters (15 fish/container). The fish were fed the experimental food twice daily at 7 am and 5 pm for 42 days.

The parameters measured in this study were the daily growth rate (DGR), specific growth rate (SGR), absolute growth rate (AGR), feed conversion ratio (FCR), feed efficiency (FE), survival rate (SR), length of the foveola gastrica, width of the foveola gastrica, and length of the intestinal villi. The biometric measurements, including the length and width of the foveola gastrica and intestinal villi, were made following the methods of Amiri *et al*. ^33^. DGR and SGR were analyzed according to the formula by Muchlisin *et al*. ^34, 35^. AGR was analyzed based on the formula of Jones ^36^:

$$\;\begin{array}{l} \text{DGR}\;\left({\text{g day}}^{\text{–1}}\right)\;\text{=}\;\left(\text{Wt – Wo}\right)\text{/t,}\\ \text{SGR}\;\left({\text{\% day}}^{\text{–1}}\right)\;\text{=}\;\left[\left(\text{Ln Wt – Ln Wo}\right)\right]\text{/t × 100,}\\ \text{AGR}\;\left({\text{cm day}}^{\text{–1}}\right)\;\text{=}\;\left(\text{L2 – L1}\right)\text{/Δt,}\\ \text{FCR = F/}\left(\text{Wt – Wo}\right)\text{,}\\ \text{FE = }\frac{\text{1}}{\text{FCR}}\;\text{× 100\%}\\ \text{Survival rate}\;\left(\%\right)=\frac{\left(Nt\right)}{No}\;\times \;100 \end{array}$$

Where Wo is initial fish weight (g); Wt is fish weight at the end of the study (g); t is the duration of the study (days). L1 is the initial length of the fish (cm), L2 is the final length of the fish (cm), Δt is the rearing period (days). Nt is the number of fish at the end of maintenance, No is the number of fish at the beginning of maintenance.

### Histological investigation and data analysis

Gastric and intestinal samples were excised at the end of the study. Four fish were taken randomly for each replication of the treatments. Fish were anesthetized in 30 ppm clove oil, and the belly of the fish was dissected following the procedure of Purushothaman *et al.* ^37^. The stomach and intestines were removed with tweezers, and placed in a bottle containing 4% formalin for 1 week. Histological sampling was carried out using the paraffin method based on Osman and Caceci ^38^. Samples were dehydrated through a gradient alcohol series (ethanol solutions of increasing concentration in 70%, 80% and 90%) and dehydrated in xylol. The stomach and intestinal samples were embedded in paraffin blocks. The paraffin blocks were cut 6-mm thick and stained with hematoxylin and eosin. The length and width of the histological preparations were measured using a binocular microscope (Zeiss Primo Star, Carl Zeiss Suzhou Co., Ltd., Beijing, China) which was projected onto a screen. Treatment of the experimental animals followed the guidelines for the use of animals in research at Syiah Kuala University (Ethic Code No. 958/2015).

The research parameters were analyzed statistically using one-way analysis of variance to detect differences followed by Duncan’s multiple range test. P < 0.05 was considered significant. All data were analyzed using SPSS software version 20 (SPSS Inc., Chicago, IL, USA). Qualitative (histological) data of the stomach and intestine were analyzed descriptively.

## Results

### Fish growth parameters

The active rice husk charcoal supplement had a significant effect on DGR and AGR, but did not significantly affect SGR ( Table 2). The highest average DGR value was observed in the 2% rice husk activated charcoal treatment, followed by 1.5% rice husk activated charcoal, the control treatment, 1% rice husk activated charcoal, 3% rice husk activated charcoal, and 2.5% rice husk activated charcoal. Furthermore, the highest average SGR value was detected in the 2% rice husk activated charcoal treatment, followed by 1.5% rice husk activated charcoal, the control treatment, 3% rice husk activated charcoal, 1% rice husk activated charcoal, and 2.5% rice husk activated charcoal. The highest average AGR value was observed in the 2% rice husk activated charcoal treatment, followed by the 1.5% rice husk activated charcoal, the control treatment, 3% rice husk activated charcoal, 1% rice husk activated charcoal, and 2.5% rice husk activated charcoal.

**Table 2.** Daily growth rate (DGR) specific growth rate (SGR), and absolute growth rate (AGR) of juvenile of giant trevally ( *Caranx ignobilis*).

Treatment		DGR Value (g day ^−1^)	SGR Value (% day ^−1^)	AGR Value (cm day ^−1^)
A.	Without supplementation of activated rice husk (0%)	0.235 ± 0.058 ^ab^	1.122 ± 0.283 ^ab^	0.068 ± 0.012
B.	Supplementation of 1% rice husk activated charcoal	0.221 ± 0.031 ^a^	1.046 ± 0.163 ^a^	0.054 ± 0.020
C.	Supplementation of 1.5% rice husk activated charcoal	0.289 ± 0,033 ^b^	1.408 ± 0.255 ^bc^	0.081 ± 0.041
D.	Supplementation of 2% rice husk activated charcoal	0.359 ± 0.065 ^c^	1.492 ± 0.325 ^c^	0.092 ± 0.018
E.	Supplementation of 2.5% rice husk activated charcoal	0.210 ± 0,018 ^a^	1.009 ± 0.126 ^a^	0.043 ± 0.009
F.	Supplementation of 3% rice husk activated charcoal	0.220 ± 0,021 ^a^	1.060 ± 0.109 ^ab^	0.058 ± 0.017

**Note:** Numbers followed by different letter superscripts are significantly different (P < 0.05).

### FCR, FE, and SR

The results showed that adding activated rice husk charcoal to feed significantly affected the FCR (P<0.001), FE (P<0.001), and SR (P<0.002) of giant trevally ( *C. ignobilis*) juveniles. The best FCR was observed in the 2% rice husk activated charcoal treatment with a value of 1.257, indicating that 1.257 kg of feed is required to increase fish weight 1 kg. The highest FCR value was detected in the 2.5% rice husk activated charcoal treatment at 1.922. Furthermore, the best FE and SR values were also observed in the of 2% rice husk activated charcoal treatment with 80.264% and 88.33%, respectively, while the lowest FE and SR values were observed in the 2.5% rice husk activated charcoal treatment at 52.114% and 65%, respectively ( Table 3).

**Table 3.** Feed conversion ratio (FCR), feed efficiency (FE), and survival rate (SR) of giant trevally ( *Caranx ignobilis*) juveniles.

Treatment		FCR	FE (%)	SR (%)
A.	Without supplementation of rice husk activated charcoal (0%)	1.638 ± 0.138 ^c^	61.381 ± 4.940 ^b^	80.00 **±** 5.443 ^bcd^
B.	Supplementation of 1% rice husk activated charcoal	1.581 ± 0.174 ^bc^	63.807 ± 6.932 ^bc^	70.00 **±**8.607 ^ab^
C.	Supplementation of 1.5% rice husk activated charcoal	1.419 ± 0.099 ^ab^	70.734 ± 4.771 ^c^	83.33 **±**6.667 ^cd^
D.	Supplementation of 2% rice husk activated charcoal	1.257 ± 0.134 ^a^	80.264 ± 9.130 ^d^	88.33 **±**3.333 ^d^
E.	Supplementation of 2.5% rice husk activated charcoal	1.922 ± 0.089 ^d^	52.114 ± 2.460 ^a^	65.00 **±**6.383 ^a^
F.	Supplementation of 3% rice husk activated charcoal	1.757 ± 0.141 ^cd^	57.198 ± 4.544 ^ab^	75.00 **±**10.000 ^abc^

**Note:** Numbers followed by different letter superscripts are significantly different (P < 0.05).

### Biometrics and histology of the foveola gastrica

**Figure 1.**
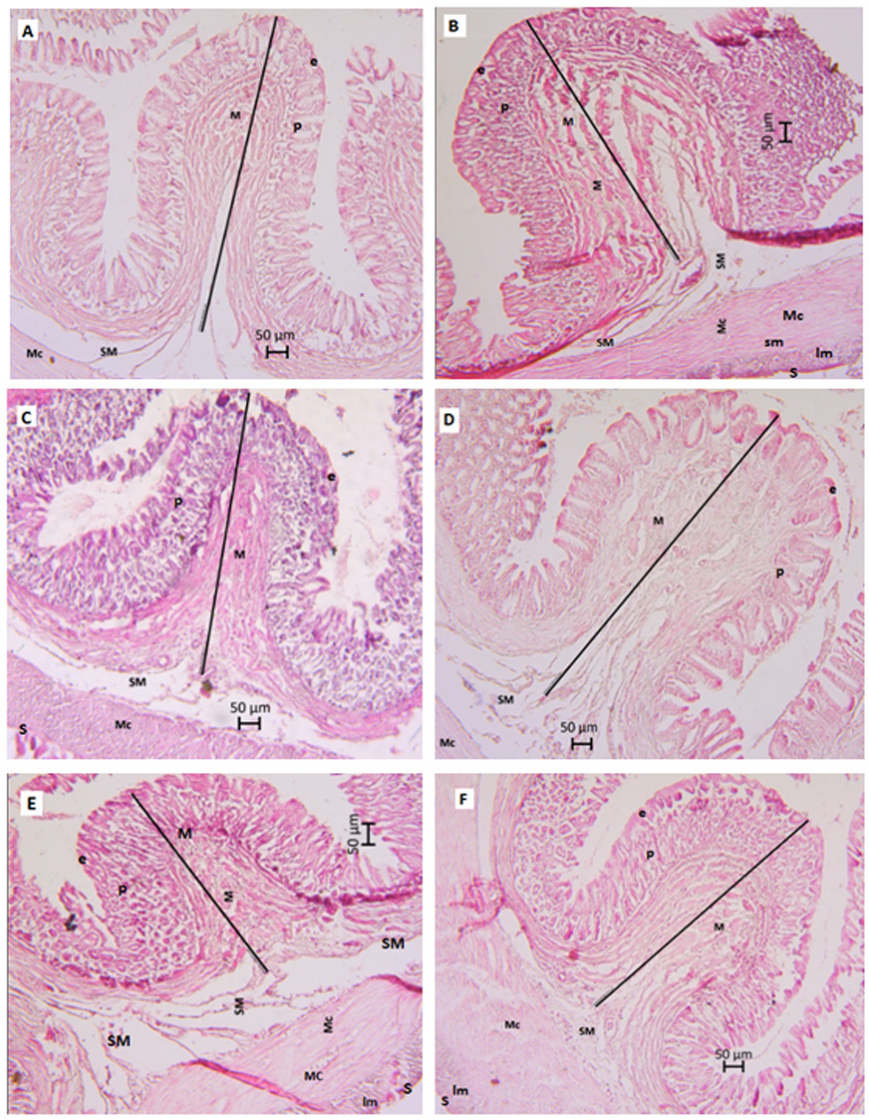
Histological sample of the giant trevally ( *Caranx ignobilis*) foveola gastrica. ( **A**) Feed without added activated charcoal (control). ( **B**) Feed with 1% rice husk activated charcoal. ( **C**) Feed with 1.5% rice husk activated charcoal. ( **D**) Feed with 2% rice husk activated charcoal. ( **E**) Feed with 2.5% rice husk activated charcoal. ( **F**) Feed with 3% rice husk activated charcoal. M, Tunica mucosa; e, epithelium lamina; p, lamina propria; SM, submucosal tunica; Mc, muscular tunica; S, serous tunica; lm, longitudinal muscles.

Adding activated rice husk charcoal to the feed had a significant effect (P < 0.05) on the length of the foveola gastrica in giant trevally ( *C. ignobilis* juveniles). The highest average length and width of the foveola gastrica were observed in the 2% rice husk activated charcoal treatment with values of 171.574 µm and 120.409 µm. respectively, while the shortest average length of the foveola gastrica was detected in the control treatment and the shortest foveola gastrica width was observed in the 1.5% rice husk activated charcoal treatment ( Table 4 and Figure 1).

**Table 4.** Average length and width of the foveola gastrica.

Treatment	The average length and width of foveola gastrica ± SD (µm)	
	Length	width
A. Without supplementation of rice husk activated charcoal (0%)	122.287 ± 10.649 ^a^	103.875 ± 16.725
B. Supplementation of 1% rice husk activated charcoal	131.583 ± 3.529 ^ab^	98.113 ± 11.229
C. Supplementation of 1,5 % rice husk activated charcoal	137.638 ± 9.665 ^ab^	89.134 ± 11.976
D. Supplementation of 2% rice husk activated charcoal	171.574 ± 27.023c	120.409 ± 10.238
E. Supplementation of 2,5% rice husk activated charcoal	147.105 ± 26.865 ^abc^	107.075 ± 28.836
F. Supplementation of 3% rice husk activated charcoal	152.787 ± 6.226 ^bc^	107.565 ± 15.729

**Note:** Numbers followed by different letter superscripts are significantly different (P < 0.05).

### Biometrics and histological intestinal villi

**Figure 2.**
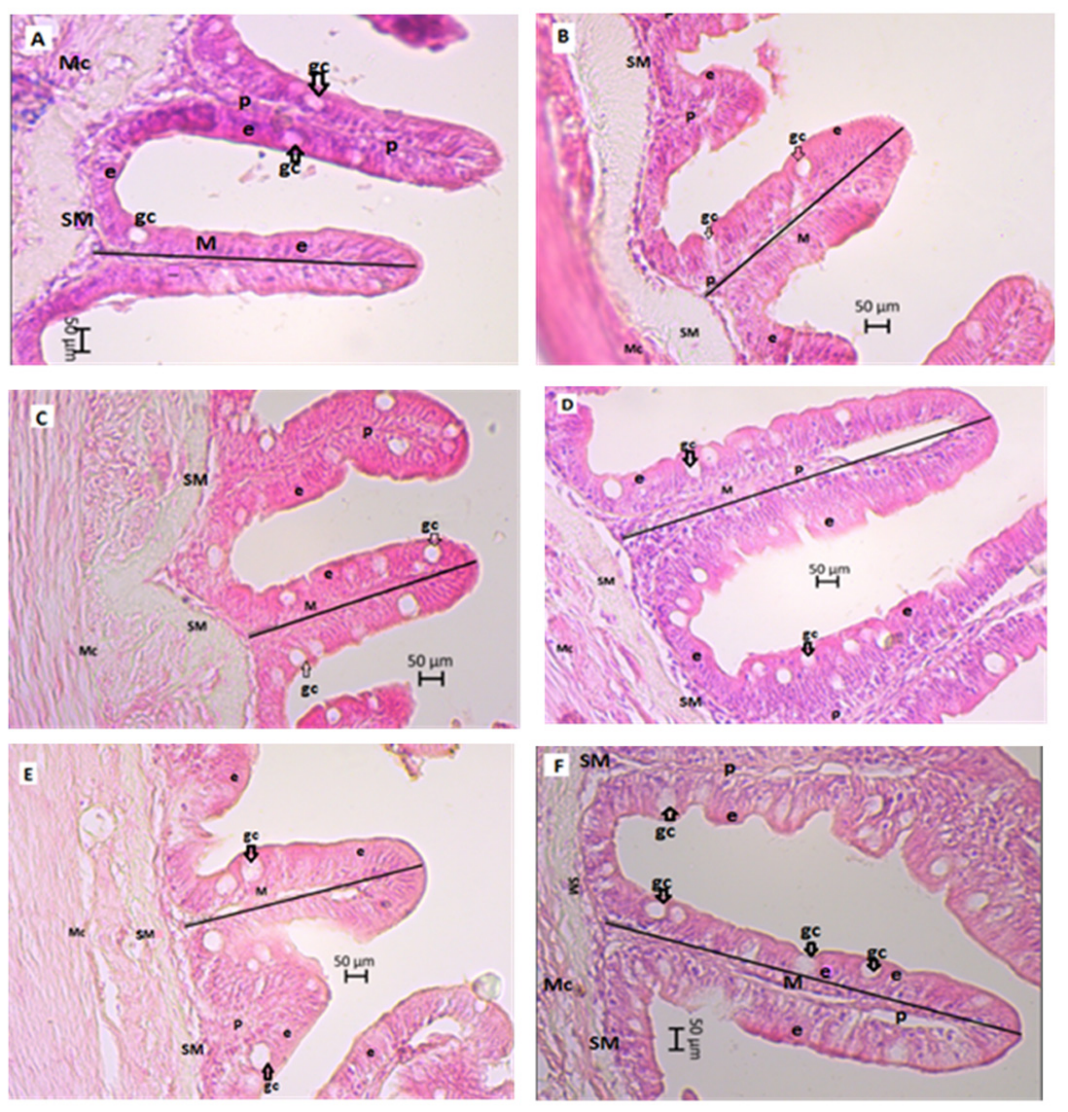
Histological samples of giant trevally ( *Caranx ignobilis*) intestinal villi. ( **A**) Feed without added activated charcoal (control). ( **B**) Feed with 1% rice husk activated charcoal. ( **C**) Feed with 1.5% rice husk activated charcoal. ( **D**) Feed with 2% rice husk activated charcoal. ( **E**) Feed with 2.5% rice husk activated charcoal. ( **F**) Feed with 3% rice husk activated charcoal. M, Tunica mucosa; SM, tunica submucosa; Mc, tunica muscularis; S, serous tunica; e, epithelial lamina; p, lamina propria; gc, goblet cell.

Supplementing with rice husk activated charcoal significantly affected (P < 0.05) the length and width of the giant trevally ( *C. ignobilis*) intestinal villi. The longest mean length and width of intestinal villi were observed in the 2% rice husk activated charcoal treatment at 64.027 µm and 16.672 µm, respectively. The shortest mean length of intestinal villi was detected in the 2.5% rice husk activated charcoal, while the shortest mean width of intestinal villi was observed in the 1% rice husk activated charcoal treatment ( Table 5 and Figure 2).

**Table 5.** Average length and width of giant trevally ( *Caranx ignobilis*) juvenile intestinal villi.

	Treatment	Average length and width of intestinal villi ± SD (µm)	
		Length	width
A.	Without supplementation of rice husk activated charcoal (0%)	56.831 ± 5.099 ^b^	13.747 ± 0.225 ^bc^
B.	Supplementation of 1% rice husk activated charcoal	55.147 ± 3.920 ^b^	11.685 ± 0.376 ^a^
C.	Supplementation of 1,5% rice husk activated charcoal	59.549 ± 0.298 ^b^	14.048 ± 0.218 ^c^
D.	Supplementation of 2% rice husk activated charcoal	64.027 ± 0.876 ^c^	16.672 ± 0.676 ^d^
E.	Supplementation of 2,5% rice husk activated charcoal	47.204 ± 0.808 ^a^	13.666 ± 0.466 ^bc^
F.	Supplementation of 3% rice husk activated charcoal	55.182 ± 3.104 ^b^	13.136 ± 0.607 ^b^

**Note:** Numbers followed by different letter superscripts are significantly different (P < 0.05).

## Discussion

Supplementing fish feed with different concentrations of rice husk activated charcoal had significant effects on DGR, AGR, FCR, FE, SR, length of the foveola gastrica, length of the villous intestine, and width of intestinal villi, but had no significant effect on SGR or the width of the foveola gastrica. The 2% activated charcoal treatment revealed the best results for all parameters, including DGR (0.359 g/day), SGR (1.492%/day), AGR (0.092 cm/day), FCR (1.257), FE (80.264%), SR (88.33%), foveola gastrica length (171.574 µm), foveola gastrica width (120.409 µm), villous bowel length (64.027 µm), and intestinal villous width (16.672 µm). These results indicate that supplementing with 2% rice husk activated charcoal was a catalyst for absorption of feed nutrients by giant trevally ( *C. ignobilis*) juveniles, which enhanced the immune system, leading to a high SR. According to Prescott *et al.* ^39^, activated charcoal functions as a bacterial endotoxin absorbent, which inhibits absorption of nutrients. In addition, Mulyono and Wibisono ^40^ reported that activated charcoal absorbs ammonia (NH _3_), which is a toxic substance. Kutlu *et al*. ^41^ stated that adding activated charcoal to feed accelerates the healing process of the mucosa by eliminating intestinal pathogenic bacteria. Furthermore, Thu *et al*. ^27^ reported that activated charcoal plays a role reducing intestinal surface pressure by removing and absorbing gases and poisons that occur along the intestine, so that nutrient absorption is optimal.

Adding more or less than 2% rice husk activated charcoal did not change the DGR, SGR or AGR values from the control treatment, indicating that increasing the rice husk activated charcoal concentration more or less than 2% is no better than control feed. However, the 1.5%–2% rice husk activated charcoal treatment increased feed protein absorption (FE) better than the other treatments, thereby reducing the quantity of feed (FCR) given to the fish. This shows that the 1.5%–2% rice husk activated charcoal treatment functioned effectively and efficiently, resulting in higher fish weights with less feed compared to the other treatments. In addition, the SR was maximum in this treatment, with a value of 83.33% in the 1.5% rice husk activated charcoal treatment and 88.33% in the 2% rice husk activated charcoal treatment. However, different results were reported by Ademola *et al.* ^29^ who found that adding 2.5% rice husk activated charcoal increases the growth and survival of catfish. This difference was likely due to the different test species, which are physiologically different.

Adding activated rice husk charcoal to the feed significantly affected the length of foveola gastrica, but did not affect the width of the foveola gastrica in giant trevally ( *C. ignobilis*) juveniles. The length of the foveola gastrica was positively correlated with increasing concentrations of rice husk activated charcoal in feed from 0%–2%, but the size decreased at concentrations of 2.5% and 3%. Although there was a decrease in the length of the foveola gastrica in the 2.5% and 3% activated rice husk charcoal treatments, the decrease was not significantly different from the longest foveola gastrica value in the 2% rice husk activated charcoal treatment. Pirarat *et al.* ^8^ reported that exceeding the optimum concentration of activated charcoal in feed does not have a positive effect on the development of digestive organs and interferes with absorption of nutrients from feed.

Figure 1 shows that the tunic muscularis forms two thin layers of muscle called the circular and the longitudinal muscles. Latania *et al*. ^42^ explained the presence and degree of muscular cooperation between the circular and longitudinal muscles indicated whether the type of feed consumed by the fishes was relatively good. This results in better absorption by the lamina epithelium and facilitates the channeling of nutrients by the lamina propria so that the foveola gastrica reacts positively by increasing in size. Furthermore, the results of histological analysis showed that giant trevally ( *C. ignobilis*) fed 2% activated charcoal developed an epithelial surface layer covering the entire foveola gastrica. This reinforces the conclusion that growth of the foveola gastrica occurred optimally with the 2% active rice husk charcoal supplement.

Table 5 and Figure 2 show that the average length and width of intestinal villi were optimum in the 2% rice husk activated charcoal treatment, indicating that activated charcoal in feed contributes to increase the length and width of the intestinal villi. Kuperman and Kuz'mina ^43^ and Mekbungwan *et al.* ^44^ reported that adding activated charcoal to feed increases the growth and intestinal function of land and aquatic animals and improves FE. Thus, growth of intestinal villi is one of the determinants of nutrient uptake during fish digestion.

## Conclusion

The results showed that supplementing the feed with different concentrations of activated rice husk charcoal significantly affected the values of DGR, AGR, FCR, FE, SR, length of foveola gastrica, length of villous intestine, and width of the intestinal villi, but had no significant effect on SGR or width of the foveola gastrica in giant trevally ( *C. ignobilis*). The 2% active rice husk charcoal treatment revealed the best results for all research parameters, including DGR (0.359 g/day), SGR (1.492%/day), AGR (0.092 cm/day), FCR (1.257), FE (FE) 80.264%), SR (88.33%), foveola gastrica length (171.574 µm), foveola gastrica width (120.409 µm), villous bowel length (64.027 µm), and villous bowel width (16.672 µm).

## Data availability

### Underlying data

Figshare: Supplementation of rice husk activated charcoal in feed and its effects on growth and histology of the stomach and intestines from giant trevally *Caranx ignobilis*, https://doi.org/10.6084/m9.figshare.12859973.v1 ^45^.

This project contains the raw data for the faveola gastica and villi of all fish examined in this study.

Figshare: Supplementation of rice husk activated charcoal in feed and its effects on growth and histology of the stomach and intestines from giant trevally *Caranx ignobilis*, https://doi.org/10.6084/m9.figshare.12860033.v1 ^46^.

This project contains uncropped, unprocessed images of the faveola gastrica of giant trevally.

Figshare: Supplementation of rice husk activated charcoal in feed and its effects on growth and histology of the stomach and intestines from giant trevally *Caranx ignobilis*, https://doi.org/10.6084/m9.figshare.12860054.v1 ^47^.

This project contains uncropped, unprocessed images of the intestinal villi of giant trevally.

### Extended data

Figshare: Supplementation of rice husk activated charcoal in feed and its effects on growth and histology of the stomach and intestines from giant trevally *Caranx ignobilis,* https://doi.org/10.6084/m9.figshare.12901784.v1 ^48^.

This project contains the daily growth rate (DGR), specific growth rate (SGR), absolute growth rate (AGR), and feed efficiency (FE) of *Caranx ignobilis*.

Figshare: Supplementation of rice husk activated charcoal in feed and its effects on growth and histology of the stomach and intestines from giant trevally *Caranx ignobilis,* https://doi.org/10.6084/m9.figshare.13094930.v2 ^49^.

This project contains the feed conversion ratio (FCR) and survival rate (SR) of Caranx ignobilis.

Data are available under the terms of the Creative Commons Attribution 4.0 International license (CC-BY 4.0).
