# Supplementary material for: Supplementation of rice husk activated charcoal in feed and its effects on growth and histology of the stomach and intestines from giant trevally, Caranx ignobilis
Source: F1000Res. 2021 Feb 15;9:1274. Originally published 2020 Oct 26. [Version 2] doi: 10.12688/f1000research.27036.2 (PMC8150121; doi:10.12688/f1000research.27036.2)
Supplement: Peer review history

# Peer review: Reviewer response for version 2

**DOI:** 10.5256/f1000research.54560.r85907

**Published:** 2021-05-25

**Authors:** Siti Azizah Mohd Nor

**Affiliations:** 1 Institute of Marine Biotechnology, Universiti Malaysia Terengganu, Terengganu, Malaysia

The manuscript entitled “Supplementation of rice husk activated charcoal in feed and its effects on growth and histology of the stomach and intestine from giant trevally, *Caranx ignobilis*” has contributed useful information for improvement of giant trevally culture through the development of a potential sustainable fish feed product. I am of the opinion that the manuscript is already worthy of indexing in its current form. Authors have generally addressed issues highlighted in previous review reports. However, having said that there are three points that I would like to suggest for authors to consider – also commented in previous review:

 Page 3. Please include at the end of the Introduction “Thus, this study aims to identify the appropriate concentration of rice husk activated charcoal supplement in fish feed which will produce optimal effect on growth parameters. We hypothesise that optimal growth can be achieved at a specific concentration”.

 Page 4. Please state the management regime to monitor (measuring pH etc) and ensure satisfactory water quality (eg waste removal) after the following sentence on this page “ The fish were fed the experimental food twice daily at 7 am and 5 pm”.

 Page 5. It would also be appropriate to add that the data was tested for homogeneity in the data analysis although it appears that they were homogeneous, based on the tabulated results.

Is the work clearly and accurately presented and does it cite the current literature?

Yes

If applicable, is the statistical analysis and its interpretation appropriate?

Yes

Are all the source data underlying the results available to ensure full reproducibility?

Yes

Is the study design appropriate and is the work technically sound?

Yes

Are the conclusions drawn adequately supported by the results?

Yes

Are sufficient details of methods and analysis provided to allow replication by others?

Yes

Reviewer Expertise:

Aquacuture and Ecological Genetics

I confirm that I have read this submission and believe that I have an appropriate level of expertise to confirm that it is of an acceptable scientific standard.

# Peer review: Reviewer response for version 2

**DOI:** 10.5256/f1000research.54560.r81267

**Published:** 2021-05-05

**Authors:** Reiny A. Tumbol

**Affiliations:** 1 Faculty of Fisheries and Marine Sciences, Sam Ratulangi University, Manado, Indonesia

The citations used on this works were closely related and were within the last ten years.

 This research was carried out accurately and meets scientific principles. The methods and data analysis used were appropriate to the type of research.

 The details of the methods and analysis used in this work were replicable, however there were some details missing. For example, the composition of the feed was not fully explained.

 The data was analysed using appropriate statistical analysis that produced reliable and accurate results.

 The conclusions were drawn from accurate data produced through appropriate methods and analysis.

 Overall, this research has been carried out in accordance with scientific principles and produced results that can be scientifically justified.

Is the work clearly and accurately presented and does it cite the current literature?

Yes

If applicable, is the statistical analysis and its interpretation appropriate?

Yes

Are all the source data underlying the results available to ensure full reproducibility?

Partly

Is the study design appropriate and is the work technically sound?

Yes

Are the conclusions drawn adequately supported by the results?

Yes

Are sufficient details of methods and analysis provided to allow replication by others?

Partly

Reviewer Expertise:

Aquaculture, fish health includes immunostimulants feed

I confirm that I have read this submission and believe that I have an appropriate level of expertise to confirm that it is of an acceptable scientific standard.

# Peer review: Reviewer response for version 1

**DOI:** 10.5256/f1000research.29862.r77124

**Published:** 2021-01-25

**Authors:** Karun Thongprajukaew

**Affiliations:** 1 Division of Health and Applied Sciences, Faculty of Science, Prince of Songkla University, Songkhla, 90112, Thailand

Based on my opinion, the manuscript entitled “Supplementation of rice husk activated charcoal in feed and its effects on growth and histology of the stomach and intestine from giant trevally, *Caranx ignobilis*” provides basic information for feed development and aquaculture of giant trevally.

 However, the manuscript writing and the presentation of data are not enough to index in its current form. I suggest the authors to use the broken-line analysis for predicting the optimal level of rice husk activated charcoal. More details and considering comments of this manuscript are listed below.

General comments:

1. Please provide common name of fish species, followed by its scientific name in parenthesis. The scientific name of species must be used the full name in the first mention and then use the abbreviations of generic name in the other places.
2. There are several errors relating to the use of capital/lowercase letters, italics, units, symbol, brackets and scientific names. Please carefully check the consistency of writing throughout the manuscript.
3. Some sections contained errors from the use of abbreviations. If all parameters were already abbreviated, so that the subsequent part must be explained using abbreviations. Please carefully check throughout the manuscript.
4. Please replace “%” by “g/kg” for feed ingredients.

Specific comments:

1. Introduction: lack of hypothesis and objective.
2. Methodology section should be re-categorized as time and site, charcoal preparation and activation, feed preparation, feeding trial (grouped experimental design, experimental fish and research parameters together), histological investigation and data analysis. In addition, it is suitable to replace the treatment titles from “A-F” to “0-3%”.
3. Please replace fish flour and shrimp flour by “fish meal” and “shrimp meal”, respectively.
4. Methods to collect uneaten feed and water management (as well as water quality) should be given.
5. How to prepare the fish before sampling? Were they starved?
6. Please provide ID or reference number for animal ethics.
7. For parameters indicating growth and feed utilization, it is necessary to provide the references for each equation; these equations are fact and all literatures are not original.
8. Table 1: 1) delete the column “protein content ingredients (%)”; 2) provide detail for the premix composition; and 3) provide proximate chemical composition of basal diet. In addition, this table must be re-written since only one feed formulation was used for all six experimental feeds. The authors should explain the formulation of “basal feed” and then included with varying levels of rice husk activated charcoal (0-3%).
9. For data analysis: Did you test the normality and homogeneity of each of your datasets? Were the data transformed before conducting the statistical analysis? In addition, I suggest the authors to use the broken-line analysis for predicting the optimal level of rice husk activated charcoal.
10. Tables 2 and 3 should be grouped as only one table, as well as for Tables 4 and 5.
11. The results section should be re-written and shortened. It is not necessary to add any value in text again since the readers can access all the values directly from tables or figures. Moreover, some result explanations were not supported by the data in the tables; please carefully check.
12. The references were not well checked and contain many errors, in both content and format. Please carefully check the references section.

Is the work clearly and accurately presented and does it cite the current literature?

Yes

If applicable, is the statistical analysis and its interpretation appropriate?

Partly

Are all the source data underlying the results available to ensure full reproducibility?

Yes

Is the study design appropriate and is the work technically sound?

Yes

Are the conclusions drawn adequately supported by the results?

Partly

Are sufficient details of methods and analysis provided to allow replication by others?

Partly

Reviewer Expertise:

Aquatic animal nutrition, digestive enzymes in aquatic animals, aquafeed chemistry

I confirm that I have read this submission and believe that I have an appropriate level of expertise to confirm that it is of an acceptable scientific standard, however I have significant reservations, as outlined above.
